# A plasma protein-based risk score to predict hip fractures

**DOI:** 10.1038/s43587-024-00639-7

**Published:** 2024-05-27

**Authors:** Thomas R. Austin, Maria Nethander, Howard A. Fink, Anna E. Törnqvist, Diana I. Jalal, Petra Buzkova, Joshua I. Barzilay, Laura Carbone, Maiken E. Gabrielsen, Louise Grahnemo, Tianyuan Lu, Kristian Hveem, Christian Jonasson, Jorge R. Kizer, Arnulf Langhammer, Kenneth J. Mukamal, Robert E. Gerszten, Bruce M. Psaty, John A. Robbins, Yan V. Sun, Anne Heidi Skogholt, John A. Kanis, Helena Johansson, Bjørn Olav Åsvold, Rodrigo J. Valderrabano, Jie Zheng, J. Brent Richards, Eivind Coward, Claes Ohlsson

**Affiliations:** 1 Cardiovascular Health Research Unit, University of Washington, Seattle, WA US; 2 Department of Internal Medicine and Clinical Nutrition, Institute of Medicine, Sahlgrenska Osteoporosis Centre, Centre for Bone and Arthritis Research at the Sahlgrenska Academy, University of Gothenburg, Gothenburg, Sweden; 3 Bioinformatics and Data Center, Sahlgrenska Academy, University of Gothenburg, Gothenburg, Sweden; 4 Geriatric Research Education and Clinical Center, VA Health Care System, Minneapolis, MN US; 5 Department of Medicine, University of Minnesota, Minneapolis, MN US; 6 Division of Nephrology, Department of Internal Medicine, Carver College of Medicine, Iowa City, IA US; 7 Iowa City VA Medical Center, Iowa City, IA US; 8 Department of Biostatistics, University of Washington, Seattle, WA US; 9 Division of Endocrinology, Kaiser Permanente of Georgia, Atlanta, GA US; 10 Charlie Norwood VAMC, Augusta, GA US; 11 Division of Rheumatology, Department of Medicine, Medical College of Georgia, Augusta University, Augusta, GA US; 12 HUNT Center for Molecular and Clinical Epidemiology, Department of Public Health and Nursing, Norwegian University of Science and Technology, Trondheim, Norway; 13 Lady Davis Institute for Medical Research, Jewish General Hospital, Montreal, Quebec Canada; 14 Quantitative Life Sciences Program, McGill University, Montreal, Quebec Canada; 15 5 Prime Sciences Inc, Montreal, Quebec Canada; 16 HUNT Research Centre, NTNU, Levanger, Norway; 17 Levanger Hospital, Nord-Trøndelag Hospital Trust, Levanger, Norway; 18 Cardiology Section, San Francisco VA Health Care System, San Francisco, CA US; 19 Department of Medicine, Epidemiology and Biostatistics, University of California San Francisco, San Francisco, CA US; 20 Department of Medicine, Beth Israel Deaconess Medical Center, Brookline, MA US; 21 Departments of Medicine, Epidemiology, and Health Systems and Population Health, University of Washington, Seattle, WA US; 22 Department of Medicine, University of California, Davis, CA US; 23 Department of Epidemiology, Rollins School of Public Health, Emory University, Atlanta, GA US; 24 Centre for Metabolic Bone Diseases, University of Sheffield Medical School, Sheffield, UK; 25 Mary McKillop Institute for Health Research, Australian Catholic University, Melbourne, Victoria Australia; 26 Department of Endocrinology, Clinic of Medicine, St. Olavs Hospital, Trondheim University Hospital, Trondheim, Norway; 27 Research Program in Men’s Health, Aging and Metabolism, Brigham and Women’s Hospital, Harvard Medical School, Boston, MA US; 28 Department of Endocrine and Metabolic Diseases, Shanghai Institute of Endocrine and Metabolic Diseases, Ruijin Hospital, Shanghai Jiao Tong University School of Medicine, Shanghai, China; 29 Shanghai National Clinical Research Center for metabolic Diseases, Key Laboratory for Endocrine and Metabolic Diseases of the National Health Commission of the PR China, Shanghai Key Laboratory for Endocrine Tumor, Shanghai Digital Medicine Innovation Center, Ruijin Hospital, Shanghai Jiao Tong University School of Medicine, Shanghai, China; 30 MRC Integrative Epidemiology Unit (IEU), Bristol Medical School, University of Bristol, Oakfield House, Oakfield Grove, Bristol, UK; 31 Department of Human Genetics, McGill University, Montreal, Quebec Canada; 32 Department of Epidemiology, Biostatistics, and Occupational Health, McGill University, Montreal, Quebec Canada; 33 Department of Medicine, McGill University, Montreal, Quebec Canada; 34 Department of Twin Research, King’s College London, London, UK; 35 Region Västra Götaland, Sahlgrenska University Hospital, Department of Drug Treatment, Gothenburg, Sweden

**Keywords:** Predictive markers, Predictive markers

## Abstract

As there are effective treatments to reduce hip fractures, identification of patients at high risk of hip fracture is important to inform efficient intervention strategies. To obtain a new tool for hip fracture prediction, we developed a protein-based risk score in the Cardiovascular Health Study using an aptamer-based proteomic platform. The proteomic risk score predicted incident hip fractures and improved hip fracture discrimination in two Trøndelag Health Study validation cohorts using the same aptamer-based platform. When transferred to an antibody-based proteomic platform in a UK Biobank validation cohort, the proteomic risk score was strongly associated with hip fractures (hazard ratio per s.d. increase, 1.64; 95% confidence interval 1.53–1.77). The proteomic risk score, but not available polygenic risk scores for fractures or bone mineral density, improved the C-index beyond the fracture risk assessment tool (FRAX), which integrates information from clinical risk factors (C-index, FRAX 0.735 versus FRAX + proteomic risk score 0.776). The developed proteomic risk score constitutes a new tool for stratifying patients according to hip fracture risk; however, its improvement in hip fracture discrimination is modest and its clinical utility beyond FRAX with information on femoral neck bone mineral density remains to be determined.

## Abstract (web summary)

The authors developed a proteomic risk score that improved the prediction of hip fractures in three validation cohorts analyzed by two different proteomic platforms. This risk score constitutes a new tool to stratify patients by hip fracture risk.

## Main

Osteoporosis is a disease related to aging, leading to an increased risk of fractures. Hip fractures are associated with significant disability and the highest mortality risk of all fracture types, with up to 20% of the patients dying in the first year following hip fracture^1,2^. By 2050, the worldwide annual number of hip fractures is expected to reach 4.5–6.3 million^2–6^.

As there are effective treatments to reduce hip fractures, identification of patients at high risk of hip fractures is important to inform efficient intervention strategies. Current available hip fracture risk calculators, such as the commonly clinically used FRAX, are algorithms that integrate the fracture risks associated with clinical risk factors with or without information on bone mineral density (BMD). According to the national guidelines in many countries, FRAX should be used to aid in fracture risk prediction to select the individuals most likely to benefit from osteoporosis treatment^7^; however, fracture risk tools such as FRAX may be improved by the addition of new biomarkers of hip fracture risk. Although it is reported that some polygenic risk scores (PRSs) for fractures and different BMD-related parameters predict hip fracture risk, none of the evaluated PRSs has yet been shown to improve hip fracture discrimination^8–11^. Furthermore, a limitation with PRSs is that these do not transfer well between different ancestral groups^12,13^.

Circulating proteins may be an alternative source of hip fracture biomarkers. Protein profiles are dynamic and may integrate information on genetic variations and environmental factors. They also reflect ongoing biological processes and may, thereby, reflect current health status and disease risk^12^. Different platforms for large-scale measurements of circulating proteins are continuously being developed to include more proteins in their analyses, including the SomaScan aptamer-based platform and the Olink double antibody proximity extension platform. Protein-based risk scores derived from either of these two platforms have recently been shown to improve prediction of some diseases of major public health importance^12,14^; however, to our knowledge, there is no report of a successful transfer of a proteomic risk score for any health condition developed using one large-scale proteomic platform to an independent validation cohort using a different proteomic platform^12^. Therefore, the present study aimed to develop a proteomic risk score to predict hip fractures, validate its performance and clinical utility in several validation cohorts and compare its performance for hip fracture prediction when using alternative proteomic platforms.

## Results

### Summary of the study design

**Fig. 1:**
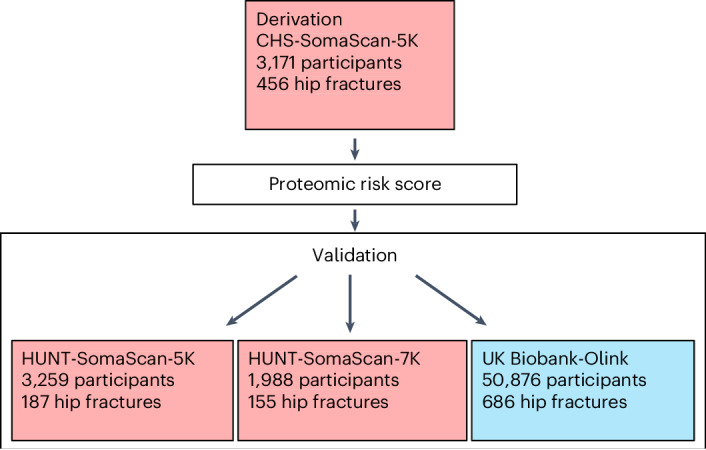
Overall design of the present study. A proteomic risk score was developed in the CHS (proteomics determined using the aptamer-based SomaScan 5K platform; 3,171 participants, 456 incident hip fractures, 39% men, mean age 74.4 years; Fig. 1 and Supplementary Table 1). The developed proteomic risk score was validated in two independent Trøndelag Health Study (HUNT) cohorts (proteomics determined using SomaScan 5K platform (3,259 participants, 187 incident hip fractures, 61% men, mean age 64.5 years) or 7K platform (1,988 participants, 155 incident hip fractures, 54% men, mean age 63.9 years)). In addition, the proteomic risk score was also validated in a subset of the UK Biobank (proteomics determined using the Olink double antibody proximity extension platform; all participants: 50,876 participants, 686 incident hip fractures, 46% men, mean age 57.0 years; randomly selected participants: 44,817 participants, 504 incident hip fractures, 46% men, mean age 56.7 years). Pink, cohorts (CHS and two HUNT cohorts) analyzed by aptamer-based SomaScan platform. Blue, cohort (UK Biobank) analyzed by the double antibody proximity extension Olink platform.

A proteomic risk score was developed in the Cardiovascular Health Study (CHS; proteomics determined using the aptamer-based SomaScan 5K platform; 3,171 participants, 456 incident hip fractures, 39% men, mean age 74.4 years; Fig. 1 and Supplementary Table 1). The proteomic risk score was validated in two Trøndelag Health Study (HUNT) cohorts (proteomics determined using SomaScan 5K platform (3,259 participants, 187 incident hip fractures, 61% men, mean age 64.5 years) or 7K platform (1,988 participants, 155 incident hip fractures, 54% men, mean age 63.9 years); Fig. 1 and Supplementary Table 1). In addition, the proteomic risk score was also validated in a subset of the UK Biobank (proteomics determined using the Olink double antibody proximity extension platform; 50,876 participants, 686 incident hip fractures, 46% men, mean age 57.0 years; Fig. 1 and Supplementary Table 1). The hip fracture prediction of the developed proteomic risk score was also compared to the hip fracture prediction of earlier developed PRSs for fractures (PRS-Fracture^15^), femoral neck-BMD (PRS-FN-BMD^16^) and the bone mass-related parameter speed of sound in the heel (PRS-gSOS^8^*)*.

### Development of the proteomic risk score in the CHS cohort

We developed three different proteomic risk scores for incident hip fractures in the CHS derivation cohort: one weighted risk score and two risk scores using the machine-learning techniques, LASSO (least absolute shrinkage and selection operator) and Elastic net, respectively (Methods and Supplementary Tables 2–4). Based on C-index improvements, the weighted proteomic risk score performed better for hip fracture prediction than the two proteomic risk scores derived by machine-learning techniques, when evaluated in two independent HUNT validation cohorts (Supplementary Tables 2–5). The weighted proteomic risk score includes 18 proteins that passed the Bonferroni-adjusted *P* value threshold of *P* < 1.0 × 10^−5^ for the association of the aptamer with incident hip fractures in the CHS cohort. The weights of the included proteins are the estimated *β* values from a Cox regression.

We next used Mendelian randomization (MR) to investigate if the proteins included in the proteomic risk score were causally related to hip fractures. We identified valid genetic instruments for 15 of the 18 circulating proteins included in the proteomic risk score^17^. None of these 15 circulating proteins displayed statistically significant evidence of a causal association with hip fractures (Supplementary Table 6), but the hip fracture genome-wide association studies (GWAS), used as source for the outcome analyses in the MR, was of limited size^5^.

### Validation of the proteomic risk score in two HUNT cohorts

**Fig. 2:**
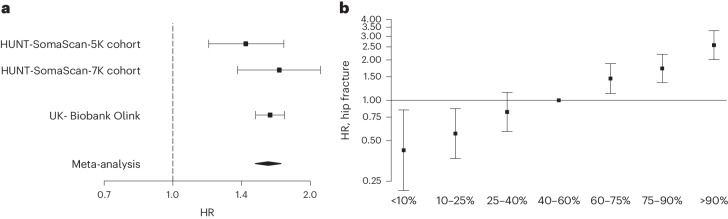
Association between the proteomic risk score and incident hip fractures. **a**, Association between the proteomic risk score and incident hip fractures in three separate validation cohorts. Base models are adjusted for age, sex and cohort-specific factors. Association with incident hip fractures is determined by Cox proportional regression models. Data are given as HRs and 95% CIs per s.d. higher risk score. The HUNT-SomaScan-5K cohort includes *n* = 3,259 participants and 187 incident hip fracture cases. The HUNT-SomaScan-7K cohort includes *n* = 1,988 participants and 155 incident hip fracture cases. The UK Biobank-Olink cohort includes *n* = 50,876 participants and 686 incident hip fracture cases. The results from the proteomic risk score were combined using fixed effects inverse-variance weighted meta-analysis with a total of *n* = 56,123 participants and 1,028 incident hip fracture cases. **b**, Associations between seven total population percentile bins of the proteomic risk score and risk of incident hip fractures in the UK Biobank. Association with incident hip fractures is determined by Cox proportional regression models adjusted for age, sex, proteomic batch, ethnicity and UK Biobank center (50,876 participants and 686 incident hip fracture cases). Data are given as HRs and 95% CIs with the 40–60% bin as reference group.

The effect sizes of this weighted proteomic risk score for hip fracture prediction were similar in the HUNT-SomaScan-5K validation cohort (Table 1), analyzed using the aptamer-based SomaScan 5K platform and in an independent HUNT validation cohort, analyzed using a later expanded version of the SomaScan platform (the SomaScan 7K platform; HUNT-SomaScan-7K cohort; Table 1, Fig. 2a and Supplementary Tables 1, 5 and 7). Next, we compared the hip fracture prediction for the proteomic risk score and available fracture/BMD-related PRSs in the two HUNT validation cohorts. Both for the two separate HUNT validation cohorts and when combined, the proteomic risk score (combined, hazard ratio (HR) 1.56; 95% confidence interval (CI) 1.36–1.79 per s.d. higher risk score) predicted hip fractures considerably more strongly than available PRSs for fractures (PRS-Fracture, HR 1.06; 95% CI 0.95–1.18), FN-BMD (PRS-FN-BMD, HR 1.20; 95% CI 1.07–1.34) and the bone mass-related parameter speed of sound in the heel (PRS-gSOS, HR 1.14; 95% CI 1.02–1.28; Table 1). In addition, the proteomic risk score, but not any of the PRSs, improved hip fracture discrimination as determined by C-index increase, starting from an age- and sex-adjusted base model, in the two HUNT validation cohorts (Supplementary Table 8).

**Table 1:** Comparison of the incident hip fracture associations for the weighted proteomic risk score and polygenic risk scores in the two HUNT cohorts and in the UK Biobank

		Cox regression				
Predictor	Number of markers	HR	95% CI	*P*	*n*	*n*_event_
HUNT-SomaScan-5K cohort						
Proteomic risk score	18	1.49	(1.24–1.80)	2.3 × 10^−5^	3,188	181
Proteomic risk score	13	1.44	(1.18–1.75)	2.4 × 10^−4^	3,188	181
PRS-Fracture	15	0.95	(0.82–1.10)	5.1 × 10^−1^	3,188	181
PRS-gSOS	21,716	1.09	(0.94–1.26)	2.7 × 10^−1^	3,188	181
PRS-FN-BMD	47	1.14	(0.98–1.32)	8.2 × 10^−2^	3,188	181
HUNT-SomaScan-7K cohort						
Proteomic risk score	18	1.66	(1.35–2.03)	9.6 × 10^−7^	1,939	153
Proteomic risk score	13	1.71	(1.38–2.12)	7.5 × 10^−7^	1,939	153
PRS-Fracture	15	1.20	(1.03–1.40)	2.2 × 10^−2^	1,939	153
PRS-gSOS	21,716	1.22	(1.04–1.44)	1.8 × 10^−2^	1,939	153
PRS-FN-BMD	47	1.28	(1.08–1.51)	3.4 × 10^−3^	1,939	153
HUNT-SomaScan combined cohorts						
Proteomic risk score	18	1.56	(1.36–1.79)	1.3 × 10^−10^	5,127	334
Proteomic risk score	13	1.56	(1.35–1.80)	1.5 × 10^−9^	5,127	334
PRS-Fracture	15	1.06	(0.95–1.18)	2.7 × 10^−1^	5,127	334
PRS-gSOS	21,716	1.14	(1.02–1.28)	1.7 × 10^−2^	5,127	334
PRS-FN-BMD	47	1.20	(1.07–1.34)	1.1 × 10^−3^	5,127	334
UK Biobank-Olink, all participants						
Proteomic risk score	13	1.63	(1.52–1.76)	7.1 × 10^−39^	50,450	679
PRS-Fracture	15	1.14	(1.05–1.23)	1.0 × 10^−3^	50,450	679
PRS-gSOS^a^	21,716	NA	NA	NA	NA	NA
PRS-FN-BMD	47	1.21	(1.12–1.31)	1.1 × 10^−6^	50,450	679
UK Biobank-Olink, randomly selected						
Proteomic risk score	13	1.64	(1.49–1.80)	1.6 × 10^−24^	44,428	501
PRS-Fracture	15	1.15	(1.05–1.26)	1.9 × 10^−3^	44,428	501
PRS-gSOS^a^	21,716	NA	NA	NA	NA	NA
PRS-FN-BMD	47	1.24	(1.13–1.35)	3.1 × 10^−6^	44,428	501

Base models were adjusted for age, sex and cohort specific factors. Associations with incident hip fractures were determined by Cox proportional regression models. A weighted proteomic risk score, including 18 proteins was developed in CHS. All 18 proteins were available in the two HUNT cohorts analyzed by the SomaScan platform, and 13 of these were available in the UK Biobank analyzed by the Olink platform. To test the replication between proteomic platforms, we also created a weighted proteomic score using the 13 proteins and evaluated its performance in the two HUNT cohorts and in the UK Biobank (using either all available participants or only the randomly selected participants). HRs and 95% CIs are given per s.d. higher risk score. Non-adjusted *P* values are derived using two-sided *z*-tests. For the analyses in Table 1, genetic analyses and proteomic analyses were required and the total number of participants and the number of incident hip fracture cases are given in Table 1. The results from the two HUNT-SomaScan cohorts were combined using fixed effects inverse-variance weighted meta-analysis.

PRS-Fracture, weighted polygenic risk score based on independent GWAS significant signals for fractures at any bone site derived from Trajanoska et al.^15^.

PRS-gSOS, polygenic risk score developed by the machine-learning technique LASSO for the bone mass-related parameter speed of sound in the heel determined by ultrasound in the UK Biobank, Lu et al.^9^.

PRS-FN-BMD, weighted polygenic risk score based on independent GWAS significant signals for FN-BMD derived from Estrada et al.^16^.

^a^The PRS-gSOS was not feasible to use in the UK Biobank as this PRS was developed in the UK Biobank.

NA, not available.

### Validation of the proteomic risk score in UK Biobank

Besides the large-scale aptamer-based proteomics platform used in the CHS derivation cohort and the two HUNT validation cohorts, the Olink double antibody proximity extension assay is an alternative large-scale proteomics platform. To avoid platform bias and enhance external validity and clinical utility, it is important to determine the transferability of proteomic risk scores between proteomic platforms.

The Olink platform was used to analyze 1,463 proteins in a subset of the UK Biobank cohort. Of the 18 proteins used in the SomaLogic-derived proteomic risk score, 13 were available and also analyzed in the UK Biobank. The majority of the participants (44,817, 88%) with proteomics data in the UK Biobank used in our analyses were selected from a randomized subset within the UK Biobank (randomly selected participants), while the remaining participants (6,059, 12%) were selected by 13 biopharmaceutical companies^18^. In the two HUNT validation cohorts, a proteomic risk score restricted to these 13 proteins predicted hip fractures similarly as the proteomic risk score including all 18 proteins (combined, HR 1.56; 95% CI 1.35–1.80; Table 1). Notably, this proteomic risk score predicted hip fractures also in the UK Biobank, when all available participants (50,876) were included, with a HR of 1.63 (95% CI 1.52–1.76) per s.d. higher risk score (Table 1). A similar effect estimate was observed when the analysis was restricted to the randomly selected participants in the UK Biobank (HR 1.64; 95% CI 1.49–1.80; Table 1). When meta-analyzing the results of the hip fracture prediction in the three validation cohorts (56,123 participants and 1,028 incident hip fracture cases), one s.d. higher proteomic risk score was associated with an HR of 1.63 (95% CI 1.52–1.74) for hip fractures (Fig. 2a and Supplementary Table 9).

The large dataset of the UK Biobank validation cohort, including a high number of incident hip fracture cases, enabled detailed analyses of hip fracture prediction. Sex- and age-stratified analyses in the UK Biobank revealed that the proteomic risk score was associated with incident hip fractures in both men and women and in both young and old participants (Supplementary Table 10). Analyses stratified on the median follow-up time of the hip fracture cases revealed that the proteomic risk score predicted both early and late hip fractures (Supplementary Table 10). Furthermore, the associations between the proteomic risk score and incident hip fractures were essentially unaltered by adjustment for grip strength or self-reported falls at baseline and only modestly attenuated by adjustment for estimated BMD in the heel using ultrasound (Supplementary Table 10). Sensitivity analyses revealed that the strength of the association was not affected by adjustment for self-reported health rating at baseline or blood sample storage time (Supplementary Table 10). Furthermore, the association remained mainly unaffected by further adjustment for body mass index (BMI), smoking, diabetes and alcohol consumption (Supplementary Table 10). Most of the participants in the UK Biobank proteomic validation cohort were self-reported as white (94%; Supplementary Table 10) and the proteomic risk score predicted hip fractures in this large group. The group of self-reported non-white participants was heterogenous and few had hip fractures (Supplementary Table 10). Exploratory analysis with limited statistical power suggested that the proteomic risk score also may predict incident hip fractures in non-white participants (Supplementary Table 10), but future larger studies are required to determine whether the developed proteomic risk score can be transferred to non-white groups.

**Extended Data Fig. 1:**
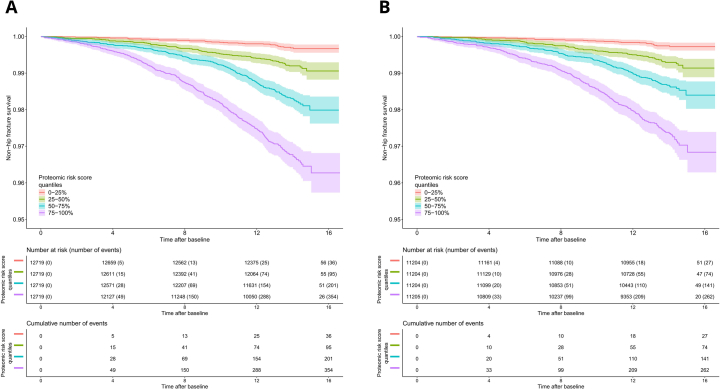
Kaplan–Meier curves of hip fractures according to proteomic risk score quartiles in UK the Biobank. Data are given as point estimates of survival rate with 95% confidence intervals. (**A**) All participants with available proteomic analyses in the UK Biobank were included (50,876 participants and 686 incident hip fracture cases). (**B**) The randomly selected participants with available proteomic analyses in the UK Biobank were included (44,817 participants and 504 incident hip fracture cases).

**Extended Data Fig. 2:**
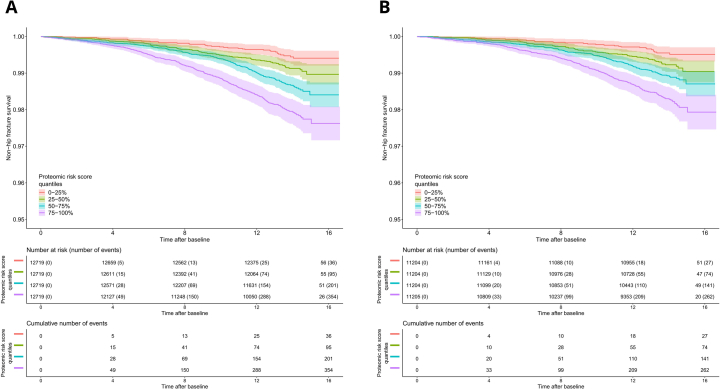
Hip fracture survival probability according to proteomic risk score quartiles adjusted for age, sex, and cohort specific factors in the UK Biobank. Data are given as point estimates of survival rate with 95% confidence intervals. (**A**) All participants with available proteomic analyses in the UK Biobank were included (50,876 participants and 686 incident hip fracture cases). (**B**) The randomly selected participants with available proteomic analyses in the UK Biobank were included (44,817 participants and 504 incident hip fracture cases).

In the UK Biobank, participants in the top 10% of proteomic risk score had an HR of 2.58 (95% CI 2.01–3.30) compared to participants with an average proteomic risk score (40–60%; Fig. 2b and Supplementary Table 11). Kaplan–Meier curves for non-hip fracture survival (Extended Data Fig. 1) and hip fracture survival probability curves (Extended Data Fig. 2) adjusted for age and sex illustrate the time dependency of the clear difference in hip fracture risk according to proteomic risk score quartiles. After 12 years, 288 participants in the highest proteomic risk score quartile but only 25 participants in the lowest proteomic risk score quartile had suffered an incident hip fracture (Extended Data Fig. 1).

Similarly, as observed in the two HUNT validation cohorts, the proteomic risk score predicted hip fracture risk more strongly than available PRSs in the UK Biobank validation cohort (Tables 1 and 2). The Pearson correlations for the proteomic risk score with PRS-Fracture (*r* = 0.013) and PRS-FN-BMD (*r* = 0.036) were low in the UK Biobank.

**Table 2:** Hip fracture discrimination for the weighted proteomic risk score and polygenic risk scores beyond CRF-FRAX and CRF-FRAX + eBMD in the UK Biobank

Base model^a^	Predictor	C-index			AUC		
		Model			Model		
		Base	Base + predictor	*P*	Base	Base + predictor	*P*
UK Biobank-Olink, all participants							
FRAX-CRF	Proteomic risk score	0.735	0.776	5.4 × 10^−9^	0.732	0.765	1.4 × 10^−7^
FRAX-CRF	PRS-Fracture	0.735	0.736	5.2 × 10^−1^	0.732	0.733	6.0 × 10^−1^
FRAX-CRF	PRS-FN-BMD	0.735	0.734	7.2 × 10^−1^	0.732	0.731	8.7 × 10^−1^
FRAX -CRF + eBMD	Proteomic risk score	0.759	0.793	8.0 × 10^−11^	0.753	0.782	9.7 × 10^−10^
FRAX -CRF + eBMD	PRS-Fracture	0.759	0.759	4.5 × 10^−1^	0.753	0.753	5.1 × 10^−1^
FRAX -CRF + eBMD	PRS-FN-BMD	0.759	0.760	5.0 × 10^−1^	0.753	0.754	4.6 × 10^−1^
UK Biobank-Olink, randomly selected							
FRAX-CRF	Proteomic risk score	0.740	0.769	2.9E × 10^−4^	0.736	0.758	1.2 × 10^−3^
FRAX-CRF	PRS-Fracture	0.740	0.742	4.3 × 10^−1^	0.736	0.738	4.5 × 10^−1^
FRAX-CRF	PRS-FN-BMD	0.740	0.738	7.0 × 10^−1^	0.736	0.734	6.8 × 10^−1^
FRAX -CRF + eBMD	Proteomic risk score	0.758	0.786	6.0 × 10^−6^	0.752	0.775	1.8 × 10^−5^
FRAX -CRF + eBMD	PRS-Fracture	0.758	0.759	1.7 × 10^−1^	0.752	0.753	1.9 × 10^−1^
FRAX -CRF + eBMD	PRS-FN-BMD	0.758	0.760	3.8 × 10^−1^	0.752	0.754	4.0 × 10^−1^

Fracture discrimination (C-index from Cox regression models and AUC from logistic regression models) beyond FRAX-CRF and/or eBMD using ultrasound in the UK Biobank. Comparisons of different AUCs were based on DeLong’s test^66^. Comparisons of different C-index were based on Kang et al.^67^. Non-adjusted *P* values are derived using two-sided *z*-tests. Genetic analyses, proteomic analyses and eBMD were required (in total 49,087 participants and 663 incident hip fractures for all participants and 43,286 participants and 487 incident hip fractures in the randomly selected participants). Results for max available follow-up time are given in this table, whereas results for follow-up time restricted to 10 years are shown in Supplementary Table 13.

FRAX-CRF, FRAX score for estimation of incident hip fracture risk using all available clinical risk factors in the UK Biobank.

PRS-Fracture, weighted polygenic risk score based on independent GWAS significant signals for fractures at any bone site derived from Trajanoska et al.^15^.

PRS-FN-BMD, weighted polygenic risk score based on independent GWAS significant signals for FN-BMD derived from Estrada et al.^16^.

^a^Base model also adjusted for sex, proteomic batch, ethnicity and UK Biobank center.

**Extended Data Fig. 3:**
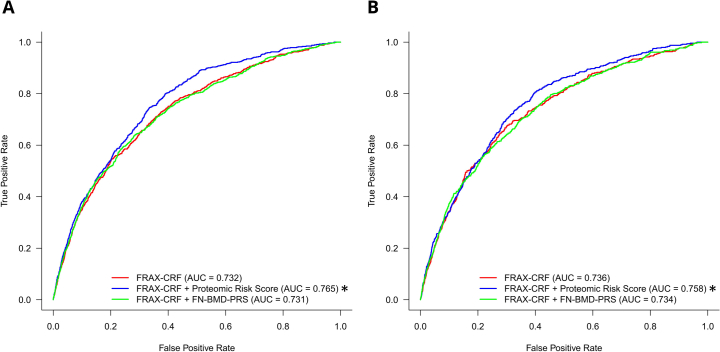
Receiver operating characteristic curves and fracture discrimination (AUC from logistic regression models) using proteomic risk score or PRS-FN-BMD beyond FRAX-CRF in the UK Biobank. Genetic analyses, proteomic analyses and eBMD were required. (**A**) All available participants (in total 49,087 participants and 663 incident hip fractures). (**B**) Randomly selected participants (in total 43,286 participants and 487 incident hip fractures). The base model was also adjusted for sex, proteomic batch, ethnicity, and UK Biobank centre. PRS-FN-BMD = Weighted polygenic risk score based on independent GWAS significant signals for femoral neck bone mineral density (FN-BMD) derived from Estrada et al.^16^. FRAX-CRF = FRAX score for estimation of incident hip fracture risk using all available clinical risk factors in the UK Biobank. The sensitivity (=true positive rate) and specificity (=1-false positive rate) are presented on the Y-axis and X-axis, respectively. *AUC for the model including FRAX-CRF + proteomic risk score (blue line) was significantly larger than the AUC for the model including only FRAX-CRF (red line). Two-sided DeLong’s test gave (**A**) for all available participants *P* = 1.4 × 10^−7^ and (**B**) for the randomly selected participants *P* = 1.2 × 10^−3^  (ref. ^18^).

Finally, we determined whether the proteomic risk score added information for hip fracture prediction in the UK Biobank validation cohort beyond the clinically used fracture algorithm FRAX, which integrates information from clinical risk factors. The proteomic risk score improved fracture prediction in models adjusted for FRAX with or without addition of estimated BMD (eBMD) (Supplementary Table 12). Discriminative analyses revealed that the proteomic risk score improved the C-index beyond FRAX, both with and without adding information on eBMD (Table 2). The proteomic risk score improved hip fracture discrimination beyond FRAX also when hip fracture discrimination was determined by the area under the curve (AUC) (Table 2 and Extended Data Fig. 3). Similar findings of improved hip fracture prediction beyond FRAX, as determined by C-index and AUC, were observed when the follow-up time was restricted to 10 years (Supplementary Table 13). In contrast, neither a PRS for fractures (PRS-Fracture) nor a PRS for FN-BMD (PRS-FN-BMD) improved hip fracture discrimination beyond FRAX (Table 2, Supplementary Table 13 and Extended Data Fig. 3). Furthermore, the proteomic risk score improved hip fracture reclassification indexes (integrated discrimination improvement, categorical net reclassification index (NRI) and categorical-free NRI) beyond a base model including FRAX or FRAX + eBMD (Supplementary Table 14). It should be emphasized that the combined NRI weights the events and non-events equally^19^. Therefore, one should rather consider the NRI results from events and non-events separately (shown in Supplementary Tables 14 and 15), showing that the improvements were mainly observed for events. Using an established clinical cutoff of 3% for hip fracture risk, above which pharmacological treatment is recommended by the Bone Health and Osteoporosis Foundation^20^, the base FRAX model correctly identified 106 true incident hip fracture cases, while the addition of the developed proteomic risk score correctly identified 198 (+87%) true incident hip fracture cases in the UK Biobank cohort (Supplementary Table 15a). A Similar magnitude of improvement in identification of true incident hip fractures was observed when the analyses were restricted to the randomly selected participants in the UK Biobank (+90%; Supplementary Table 15b). These improvements were due to improved sensitivity (Supplementary Table 15c,d).

In contrast, minor improvements of the different reclassification indexes were observed for PRS-FN-BMD when starting from a base FRAX model but not when starting from a base FRAX + eBMD model (Supplementary Table 14). PRS-Fracture did not improve fracture reclassification starting from a base model including FRAX or FRAX + eBMD (Supplementary Table 14).

### Association with fractures at different bone sites

Next, we evaluated the performance of the developed hip fracture proteomic risk score for prediction of fractures at other bone sites besides hip fractures in the UK Biobank. Although, the proteomic risk score was associated with incident fractures at all bone sites evaluated, the association of the proteomic risk score was substantially stronger with hip fractures (HR 1.64, 95% CI 1.50–1.81) than with non-hip fractures such as forearm fractures (HR 1.18, 95% CI 1.08–1.29) and lower leg fractures (HR 1.20, 95% CI 1.08–1.33; Supplementary Table 16).

## Discussion

Patients at high risk of fractures at the hip, the clinically most important fracture site, are important to identify early to start effective interventions. We developed a proteomic risk score that improved hip fracture prediction and discrimination in three independent validation cohorts, analyzed by two substantially different proteomic platforms. The developed proteomic risk score predicted hip fractures similarly in both young and old participants, and in both men and women. Finally, when added to FRAX with or without information on eBMD, the proteomic risk score, but not available PRSs, improved hip fracture discrimination. These results show that the proteomic risk score is strongly predictive of incident hip fractures in multiple independent populations.

Several PRSs have been developed that primarily predict different measures of BMD and some of these also predict hip fracture risk^8–11^; however, none of these PRSs has been shown to improve hip fracture discrimination as determined by C-index or AUC beyond FRAX estimates, limiting their clinical utility^8–11^. Similarly, none of the available fracture or BMD-related PRSs improved hip fracture discrimination in any of the three validation cohorts in the present study. In contrast, the proteomic risk score improved hip fracture discrimination, and the associations with incident hip fractures were substantially stronger for the proteomic risk score than for the different available fracture and BMD-related PRSs.

Similar to a recent study evaluating risk of coronary artery disease, weak correlation was found between available PRS and proteomic risk score in the present study evaluating hip fracture risk^21^. The low correlation between proteomic risk score and PRS in both studies suggests that genetics and proteomics may contribute independent information for prediction of outcomes. In the recent study on coronary artery disease, genetics and proteomics added complementary information to the clinical risk factors for prediction of coronary artery disease^21^. In contrast, the developed proteomic risk score, but not available PRSs, improved hip fracture discrimination beyond clinical risk factors in the present study. As proteins integrate the effects of genes with effects caused by the environment, age, comorbidities, behaviors and drugs, the circulatory proteomic profile can provide information about health status and disease risk^22,23^, most likely explaining why the proteomic risk score improved hip fracture discrimination more efficiently than available PRSs. We also note that current PRSs are based on relatively small samples of fractures, most of which occur at an age when the heritability of fracture is reduced^24^. This is supported by previous studies demonstrating that the available PRSs display rather modest performances for hip fracture discrimination^8–11^. One of the proteins, CD14, included in our proteomic risk score (Supplementary Table 2) has previously been reported to be associated with incident hip fractures in the MrOS cohort^25^. We recently performed a large-scale meta-analysis of the association between circulating proteins, measured by the aptamer-based technique, and hip fracture risk and identified 23 hip fracture signals^26^. Fifteen of these signals correspond to proteins included in the proteomic risk score developed in the present study.

As proteins integrate the effects of genes with effects caused by the environment, age, comorbidities, behaviors and drugs, the circulatory proteomic profile can provide information about health status and disease risk, most likely explaining why the proteomic risk score improved hip fracture discrimination more efficiently than available PRSs. We believe that many of the circulating proteins included in the identified proteomic risk score may be markers of current health status and the biological age, which in turn has an impact on hip fracture risk. This notion is supported by a recent report demonstrating that four of the circulating proteins in our proteomic risk score (GDF15, MMP12, EGFR and WFDC2) also are included in a proteomic aging clock score that predicts accelerated biological aging and several age-related outcomes after adjusting for chronological age^27^. Based on these findings, we propose that these four proteins predict hip fracture risk because they are general markers of biological aging and hip fracture risk increases when biological age increases. Future studies are warranted to determine what proportion of the plasma proteins included in the proteomic risk score are causally related to hip fractures and their underlying mechanism. Nevertheless, the clinical utility of a protein-based risk score for hip fracture prediction does not depend on whether the included proteins are causally related to hip fractures.

It is not only important to validate the performance of a developed proteomic risk score in independent validation cohorts, but also that it can be transferred to alternative proteomic platforms. Currently, there are two main proteomic platforms used in biomedical research: the SomaScan aptamer-based platform and the Olink double antibody proximity extension platform. We developed the proteomic risk score using the aptamer-based platform in CHS and successfully validated its performance to predict hip fractures in two independent HUNT cohorts, using two different versions of the SomaScan aptamer-based platform. Notably, the proteomic risk score was also validated in the UK Biobank where the circulating proteome was analyzed using the substantially different Olink double antibody proximity extension platform. To our knowledge, the present study is the first to report of a successful transfer of a proteomic risk score for disease prediction from one large-scale proteomic platform to an independent validation cohort analyzed by an alternative proteomic platform^12^.

As clinically used hip fracture risk tools such as FRAX may be improved by the addition of validated new biomarkers, we determined the clinical utility of the proteomic risk score beyond FRAX with or without information on eBMD in the large UK Biobank validation cohort. The proteomic risk score significantly improved the hip fracture discrimination and reclassification indexes beyond a base model, including both FRAX without and with addition of eBMD. Based on these findings, we propose that the proteomic risk score is a biomarker candidate to be included as a new risk marker in future updates of FRAX^28^. Notably, the proteins included in the proteomic risk score can either be analyzed by the SomaScan aptamer-based platform or by the Olink double antibody proximity extension platform using the same proteomic risk score as described in Supplementary Table 2, yielding similar performance for hip fracture prediction. This enhances the accessibility of the proteomic risk score for clinical use.

Among the three developed proteomic risk scores in CHS, the weighted proteomic risk score version, predicted hip fractures best when evaluated in two HUNT validation cohorts. The inferior performance of the two proteomic risk scores developed using the machine-learning techniques LASSO and Elastic net may be due to overfitting of these proteomic risk scores in the CHS derivation cohort.

The average baseline age and average age of incident hip fracture for the participants in the CHS derivation cohort were higher than for the participants in the three validation cohorts. The participants in the UK Biobank validation cohort were younger than the participants of the two HUNT validation cohorts. For the validation cohorts, the average ages at incident hip fractures were slightly lower compared to the average age of hip fracture cases in the general population^29^. As the effect estimates for the association between the developed proteomic risk score and incident hip fractures were similar for the different evaluated cohorts, the proteomic risk score seems robust to use for hip fracture prediction within a wide age range. This notion is further supported by the similar effect estimates observed for the younger and older participants in our age-stratified analyses in UK Biobank; however, further studies are required to validate the performance of the proteomic risk score in older people.

Although the proteomic risk score was associated with incident fractures at all bone sites evaluated, the association of the proteomic risk score was substantially stronger with hip fractures than with non-hip fractures such as forearm fractures and fractures at the lower leg. This bone-site specificity is probably due to the fact that the proteomic risk score was developed to predict hip fractures and that the risk factors for fractures at different bone sites partly differ.

The strengths of the present study include the high number of incident hip fractures (*n* = 456 in the derivation cohort and *n* = 1,028 combined in the three validation cohorts) and the use of three independent validation cohorts analyzed by two different proteomic platforms.

The present study has several limitations. The group of non-white individuals in the UK Biobank was heterogenous, defined using self-reported information and had few hip fractures. Although our exploratory analyses suggested that the proteomic risk score may predict incident hip fractures in non-white participants, future larger studies are required to determine if the developed proteomic risk score can be transferred to non-white groups. Thus, we call for caution on generalizing the findings beyond the populations studied in the present study. In addition, we did not adjust our analyses for kidney function, which could serve as a confounder in our analyses; however, we used median-normalized data across all aptamers, which attenuates kidney function associations and thus partly resembles adjustment for eGFR^30,31^. Another limitation is that information on FN-BMD was not available for the included participants at the time of the proteomic baseline sample in the UK Biobank; however, information on eBMD in the heel, a strong predictor of hip fractures^32,33^, was available for most of the included participants at the time of the baseline samples used for proteomic analyses. Therefore, eBMD was used as a hip fracture-related BMD measure in some of the models determining the clinical utility of the proteomic risk score beyond FRAX. Further studies in cohorts with information on circulating proteomics, FRAX estimates, and FN-BMD measures at baseline are warranted to determine the clinical utility of the developed proteomic risk score beyond FRAX with information on FN-BMD. Finally, it should be emphasized that the current costs for running the complete SomaScan or Olink proteomic assays, used in the present study, are substantial. Presently, it is not established that the magnitude of excess hip fracture risk predicted by the proteomic risk score is great enough to distinguish between individual patients for treatment decisions that will be cost-effective; however, future targeted hip fracture proteomics panels, based on the present findings, may be cheaper and add cost-effective information to future improved updates of FRAX^28^.

In conclusion, the developed proteomic risk score enhanced hip fracture prediction and discrimination in three separate validation cohorts analyzed by two substantially different proteomic platforms. When added to FRAX with or without information on eBMD, the proteomic risk score, but not available PRSs, improved fracture discrimination. We propose that the developed proteomic risk score is a biomarker candidate to be included as a new risk marker in future updates of the fracture prediction tool FRAX^28^. The developed proteomic risk score constitutes a new tool for stratifying patients according to hip fracture risk; however, its improvement in hip fracture discrimination is modest and its clinical utility beyond FRAX with information on FN-BMD remains to be determined.

## Methods

### Cohorts

The CHS was used to derive proteomic risk scores for hip fractures whereas two subcohorts within the HUNT study and a subsample of the UK Biobank were used for validation of the identified proteomic risk scores (Fig. 1).

### The Cardiovascular Health Study

The CHS is a population-based longitudinal study of cardiovascular disease in older people (>65 years of age) recruited from four US communities^26,34^. Fasting EDTA-plasma was collected in 1992–1993 and was stored at −70 °C until used for proteomic profiling^26^. Incident hip fractures after the 1992–1993 study visit through 2015 were identified from hospital discharge diagnosis codes. The 1992–1993 exam was attended by 5,265 participants^26^. Of those participants, all 3,171 with unthawed plasma available in 2020, had such plasma used for proteomic profiling using a SomaScan aptamer-based platform (5K SomaScan v.4.0 assay)^35,36^. The CHS study was approved by institutional review boards at each of the four field centers and the Coordinating Center. The CHS is currently under a single institutional review board at the University of Washington (current approval no. MODCR00000825). All CHS participants provided written informed consent. No compensation was provided to participants.

### The Trøndelag Health Study

HUNT is a longitudinal health study in the Norwegian county of Trøndelag and it includes data from four visits between 1984 and 2019 (refs. ^26,37,38^).

The HUNT-SomaScan-5K cohort is a subcohort consisting of 3,259 participants from a HUNT cardiovascular project, including 1,270 participants with incident cardiovascular events and 1,989 participants without incident cardiovascular events^26^. Therefore, we adjusted all HUNT-SomaScan-5K association analyses for incident cardiovascular disease (yes/no). The HUNT-SomaScan-7K cohort is a subcohort consisting of 1,988 new participants from two more recent studies, focusing on venous thromboembolism^39^ and abdominal aortic aneurysm^40^. We adjusted HUNT-SomaScan-7K association analyses for incident venous thromboembolism (802 cases) and incident abdominal aortic aneurysm (232 cases). The HUNT study has ethical approval from the Regional Committee for Medical and Health Research Ethics (REK Central Norway 2015/615) and informed consent was obtained from all participants^26^. No compensation was provided to participants.

Previously unthawed, non-fasting, EDTA-plasma samples from the HUNT3 visit (2006–2008; stored at −80 °C) were used for proteomic profiling. For the HUNT-SomaScan-5K cohort (using the same aptamer-based SomaScan platform as used in CHS) proteomic analyses were performed in 2017 (ref. ^26^), whereas the analyses for the HUNT-SomaScan-7K cohort (using the extended aptamer-based SomaScan 7K platform (v.4.1)) were performed in 2022.

### The UK Biobank

The UK Biobank is a population-based cohort of approximately 500,000 participants aged 37–73 years. The participants were recruited between 2006 and 2010. Participant data include genome-wide genotyping, exome sequencing, whole-body magnetic resonance imaging, electronic health record linkage, blood and urine biomarkers and physical and anthropometric measurements^18^. For proteomic analyses, EDTA-plasma was collected and stored at −80 °C until samples were analyzed. The plates were kept at −80 °C and then sent to Olink (Uppsala, Sweden) for proteomic profiling. The UK Biobank Pharma Proteomics Project (UKB-PPP) is a collaboration between the UK Biobank and 13 biopharmaceutical companies^18^. Using baseline samples, 50,876 participants with successful proteomic analyses (starting in 2021) and available information on covariates (age, height, sex, weight and ethnicity) and not included in the COVID-19 case–control imaging study were included in the present proteomic hip fracture study. The majority (44,817, 88%) of the included participants were selected from a randomized subset within UK Biobank (randomly selected participants), while the remaining participants were selected by the 13 biopharmaceutical companies^18^. In sensitivity analyses, we restricted our analyses to the participants in the large randomized subsample, showing an essentially unchanged effect estimate for the association between the proteomic risk score and hip fractures in UK Biobank compared to the effect estimate when including all participants. Further details are available at https://biobank.ndph.ox.ac.uk. The UK Biobank has ethical approval from the North West Multi-centre Research Ethics Committee (North West Research Ethics Committee, 11/NW/0382) and informed consent was obtained from all participants. No compensation was provided to participants. The present research was approved by the UK Biobank Research and Access Committee (application no. 51784).

#### FRAX estimates in the UK Biobank

FRAX estimates of the 10-year probability of experiencing a hip fracture for the participants in the UK Biobank were calculated by the international FRAX team (J. A. Kanis and H. Johansson) using the UK-specific FRAX tool (https://www.sheffield.ac.uk/FRAX/; v.1.4.4.) incorporating clinically relevant risk factors. Clinically relevant risk factors included in the FRAX algorithm were measured at the baseline visit for the UK Biobank such as age, sex, BMI (in kg m^−2^), previous fractures (hip fractures and other osteoporotic fractures), current smoking, glucocorticoid use, rheumatoid arthritis and diagnosis of secondary causes of osteoporosis (type 1 diabetes, osteogenesis imperfecta in adults, untreated long-standing hyperthyroidism, hypogonadism or premature menopause, chronic malnutrition, chronic malabsorption and chronic liver disease). Participants with missing information on any FRAX clinical risk factor were considered free of the corresponding risk factors for the derivation of FRAX probability, as suggested by the FRAX model (https://www.sheffield.ac.uk/FRAX/faq.aspx). The relationships between risk factors and fracture risk in the FRAX model have been constructed using information derived from the primary data of population-based cohorts from around the world, including centers from North America, Europe, Asia and Australia, based on a series of meta-analyses^41–47^. The FRAX algorithm has been externally validated with a similar geographic distribution with a follow-up in excess of 1 million patient-years and its construct summarized in a World Health Organization technical report^48,49^. Since its launch in 2008, the FRAX model has proven to be well calibrated in diverse populations from Canada, Israel, Japan, Norway, Taiwan and the United Kingdom^50–55^.

#### eBMD using ultrasound in the UK Biobank

Quantitative ultrasound of the heel was used to obtain a noninvasive eBMD that predicts fracture^32,33^. A Sahara Clinical Bone Sonometer (Hologic Corporation) was used for quantitative assessment of calcanei in UK Biobank participants. Details of the complete protocol are publicly available on the UK Biobank website (www.ukbiobank.ac.uk/). eBMD (g cm^−2^) was derived as a linear combination of speed of sound and bone ultrasound attenuation (eBMD = 0.0025926 × (bone ultrasound attenuation + speed of sound) − 3.687)^56^. Lower eBMD predicted high risk of incident hip fractures also in the present UK Biobank proteomics cohort (HR 1.76; 95% CI 1.60–1.93, per s.d. lower eBMD; the model was adjusted for age, sex, height, weight, ethnicity and assessment center).

### Incident hip fractures

Hospitalizations were self-reported by CHS participants (every 6 months) and hospitalizations not reported by participants were identified from Medicare claims data^26^. In the CHS, incident hip fractures were identified from hospital discharge International Classification of Diseases, Ninth Revision (ICD9) codes 820.xx, covering the time following the 1992–1993 CHS study visit through 2015. Pathologic fractures (ICD9 code 773.1x) and fractures caused by motor vehicle accidents (E810.xx–E825.xx) were excluded^26^.

Hospital-based registries in the region were used to collect hip fracture data for the HUNT participants, covering the time interval from baseline (HUNT3 survey in 2006–2008) until March 2021. The following ICD10 codes S72.0, S72.1 and S72.2 or ICD9 code 820 were used for hip fracture definition^26^.

In the UK Biobank, hip fractures were identified using ICD10 codes S72.0, S72.1 and S72.2 or ICD9 code 820 derived from registries covering the interval from baseline samples 2006–2010 until 31 October 2022.

### Proteomics

#### SomaScan

The SomaScan 5K v.4.0 (CHS cohort and HUNT-SomaScan-5K cohort) and the SomaScan 7K v.4.1 (HUNT-SomaScan-7K cohort) aptamer-based assays were used to measure the concentrations of plasma proteins. The concentrations are given as relative fluorescent units. In brief, the aptamers from SomaScan are single-stranded DNA-based reagents called SOMAmers (slow off-rate modified aptamers). The negatively charged SOMAmers are designed to be complementary to the shape of the natively folded target proteins and bind the target protein tightly and specifically at a ratio of 1:1 (refs. ^35,36^). The method, which takes advantage of new chemically modified nucleotides, converts the measurement of protein levels into the measurement of nucleic acid levels assessed by a DNA oligo-array plate reader^35,57^. The assay sensitivity has a median lower limit of detection in the femtomolar range, which is comparable to that of typical immunoassays^14,58^. Results of these assays, reported in relative fluorescent units, are approximately proportional to plasma protein concentrations. Median intra- and inter-assay coefficients of variation for SomaScan v.4.0 and 4.1 are low at approximately 5% (refs. ^14,31,58^). The assays used for these analyses include 5,284 aptamers for the 5K platform and 7,596 aptamers for the extended 7K platform. We excluded aptamers marked as ‘deprecated’ (indicating a retired aptamer) and those marked as ‘non-human’ from the present analyses. Samples flagged by SomaLogic for poor quality assay were also removed. After these exclusions, 4,979 aptamers were available for analyses in the 5K platform and the same aptamers were also evaluated in the 7K platform.

#### Olink

The samples from UK Biobank were analyzed using the Olink Explore 1536 platform, measuring 1,472 protein analytes corresponding to 1,463 unique proteins. The Olink platform is considered a specific antibody-based assay. In brief, Olink uses a proximity extension assay technology where complimentary DNA-tags on matched pairs of antibodies hybridize when the antibodies are bound to the same target protein (antigen), enabling DNA amplification of the protein signal with a DNA polymerase. The PCR product is quantified and detected on a next generation sequencing readout^59,60^. The Olink Explore platform consists of four panels focusing on inflammation, oncology, cardiometabolic and neurology proteins. Each panel has 12 internal controls and three proteins (CXCL8, IL-6 and TNF) are included in all four panels for quality assurance purposes. The performance of each protein assay is validated based on specificity, sensitivity, dynamic range, precision, scalability detectability and endogenous interference^61^. The median intra-individual coefficient of variation was 6.3%. The mean correlations across different panels for each of the three proteins (CXCL8, IL-6 and TNF) measured on all four protein panels varied between *r* = 0.81 and 0.96.

### Statistical analyses

#### Proteomic risk scores

##### Weighted proteomic risk score

From the Cox regression of the associations between each of the 4,979 aptamers and incident hip fractures, a weighted proteomic risk score was developed in the CHS derivation cohort. The proteomic risk score includes the 18 aptamers passing the Bonferroni-adjusted *P* value threshold of *P* < 1.0 × 10^−5^ in the CHS cohort (Supplementary Table 2). The weights are the estimated *β* values from the Cox regression.

##### LASSO proteomic risk score

We used LASSO with repeated data splitting to identify top aptamers (proteins) for inclusion in a risk score, using as few proteins as possible in the model. Protein prediction models were derived in CHS with the R package ‘glmnet’. For Cox regression analysis with LASSO penalty, a tenfold cross-validation was carried out for tuning parameter selection. The CHS data were randomly split 500 times, with 70% of the data in each split used as training data for model fitting and 30% of the data used for model testing. Age, sex and race were forced into the model, while log-transformed and normalized protein values for all available aptamers were included as parameters. For each of the 500 splits, a set of proteins relevant for prediction were identified by the model based on the λ value, which gives the minimum mean cross-validated error in our model. Based on results from the 500 data splits, proteins were ranked by the frequency with which they were selected by the LASSO model.

The top 1–30 aptamers, ranked by their average selection frequency and coefficient estimates using LASSO, were carried into usual Cox regression models via repeated sample splitting. The data were split ten times, again into 70% training and 30% testing data, with C-index and AUC calculated for models containing age, sex, race and between 1–30 aptamers, by selection frequency in the previous step. A parsimonious model of top aptamers was chosen based on the number of aptamers included in the model that maximized AUC and C-index. The model that was found to be the most reliable protein model for prediction of fractures included 22 proteins and was validated in the HUNT cohorts (Supplementary Table 3).

##### Elastic net proteomic risk score

Using similar methods as for the LASSO machine-learning proteomic risk score, additional analyses were conducted using an elastic net (EN) penalty. This method is a hybrid of ridge regression and LASSO regularization which performs well in the setting of multicollinearity, in which parameters are highly correlated, as is seen in large proteomics datasets. In our EN analyses, an *α* value of 0.9 was chosen to allow for some multicollinearity while generating a parsimonious model, the top model, including 20 proteins (Supplementary Table 4).

#### Polygenic risk scores

We selected PRSs for fractures (PRS-Fracture^15^), FN-BMD (PRS-FN-BMD^16^) and the bone mass-related parameter speed of sound in the heel (PRS-gSOS^8^) based on the previously published largest GWAS^8,15,16^, yielding the highest number of independent loci for each phenotype (see details below). The performances of PRS-FN-BMD and PRS-gSOS for fracture prediction have been published previously^8–11^. There is also an alternative BMD-based PRS evaluating fracture prediction^62–64^, which we have not used, as it is based on an early publication with a considerably smaller GWAS for FN-BMD^65^.

*PRS-Fracture* The weighted PRS was based on 15 independent GWAS significant signals for fractures at any bone site derived from a previous fracture GWAS^15^.

*PRS-FN-BMD* The weighted PRS score was based on 47 independent GWAS significant signals for FN-BMD^16^.

*PRS-gSOS* The PRS was developed by the machine-learning technique LASSO using 21,717 genetic markers for ultrasound-derived speed of sound in the heel in the UK Biobank^8^.

#### Cox proportional hazards models

For hip fracture survival analyses, HRs, 95% CIs and significance levels were calculated using Cox proportional hazards models. Fractures were assessed from baseline to the diagnosis of fracture, death or the end of follow-up, whichever occurred first. The HR for a proteomic risk score or PRS was reported per one s.d. higher risk score. In addition, exploratory stratified analyses according to sex, age and ancestry were performed in the large UK Biobank cohort. Hip fracture risk in different total population percentile bins of the proteomic risk score, compared to participants with an average proteomic risk score (40–60%), was also determined.

#### Time-dependent analyses

Kaplan–Meier curves and hip fracture survival probability curves adjusted for age and sex were used to explore the time dependency of the difference in hip fracture risk according to proteomic risk score quartiles.

#### Fracture discrimination

A receiver operating characteristic (ROC) AUC was calculated using the roc.test function in the pROC R package. The difference between AUCs were tested using DeLong’s test, in the same R function (https://cran.r-project.org/web/packages/pROC/index.html)^66^.

C-index and 95% CIs were calculated using the rcorr.cens function in the Hmisc R package (F. E. Harrel proposed the method and wrote the R package; https://cran.r-project.org/web/packages/Hmisc/index.html). Differences between C-index were tested using the compareC function in the R package compareC (https://cran.r-project.org/web/packages/compareC/index.html)^67^.

#### Fracture reclassification

To evaluate the improvement in reclassification gained by adding a variable to a baseline predictor, the NRI using 3% predicted hip fracture threshold (NRI categorical; above which pharmacological treatment is recommended by the Bone Health and Osteoporosis Foundation^20^), the integrated discrimination improvement and the category-free NRI were used^68,69^.

All statistical computations were performed using R.

### Association with fractures at different bone sites

We also evaluated the performance of the developed hip fracture proteomic risk score for prediction of fractures at other bone sites besides hip fractures in the UK Biobank. Fracture cases were identified using ICD10 and ICD9 codes (Supplementary Table 16) and included the following fracture groups: forearm fractures, hip fractures, major osteoporotic fractures (includes distal forearm fractures, hip fractures, vertebral fractures and upper arm fractures) and fractures of the lower leg.

### Phase 2 proteins in the UK Biobank

During the revision of this manuscript, the phase 2 proteins from the Olink platform were released in a subsample of the participants included in the UK Biobank validation cohort. This enabled the inclusion of three additional proteins, resulting in a proteomic risk score with 16 proteins instead of 13 in this subsample of UK Biobank (Supplementary Table 2); however, the strengths of the association per s.d. increase in the proteomic risk score were very similar for the originally designed proteomic risk score of 13 and the new proteomic risk score of 16 in this subsample of the UK Biobank (*n* = 39,551, proteomic risk score of 13, HR 1.70, 95% CI 1.54–1.87; proteomic risk score of 16, HR 1.70, 95% CI 1.54–1.88). As the effect sizes for the hip fracture associations for proteomic risk score of 13 and proteomic risk score of 16 were similar and the sample size for proteomic risk score of 13 was substantially larger, the data in the article are presented using the originally designed proteomic risk score of 13.

### Mendelian randomization

Genetic instruments for the plasma proteins included in the proteomic risk score were selected from a previously published GWAS on circulating proteins analyzed by the SomaScan aptamer-based technique in 35,559 Icelanders^17^. Correlated single-nucleotide polymorphisms were removed using LD-pruning with an *r*^2^ threshold of 0.01. Outcome results were selected from a published GWAS on hip fractures (11,516 hip fracture cases)^5^. MR analyses were performed using the MendelianRandomization R package. Causal associations were estimated using either Wald ratio or inverse-variance weighted fixed effects depending on the number of valid genetic instruments.

### Statistics and reproducibility

No statistical test was used to predetermine the sample sizes, but our sample sizes are similar to those reported in previous publications using proteomic data from the cohorts used in the present study^18,26^. The numbers of included participants for each analysis are given in the table and figure legends. We used population-based longitudinal cohorts and no randomization was performed by us; however, in the UK Biobank the subcohort selected for the proteomic analyses was previously randomly selected to be representative of the whole UK Biobank cohort as described in a previous publication^18^. To reduce potential selection bias, participants selected for the UK Biobank COVID-19 study were excluded from the present study evaluating proteomic risk scores^18^. The investigators were not blinded to allocation during experiments and outcome assessment. Data collection (incident hip fractures) and proteomic analyses were performed before the initiation of the present study. The statistical tests used are given in the legends of all tables and figures. The main finding that the weighted proteomic risk score predicted hip fracture risk was observed in three independent validation cohorts. Data distribution was assumed to be normal, but this was not formally tested. We followed the STARD guidelines.

### Reporting summary

Further information on research design is available in the Nature Portfolio Reporting Summary linked to this article.

## Supplementary information

Supplementary Information STARD guidelines.

Reporting Summary

Supplementary Table Supplementary Tables 1–16.

Supplementary Code Custom written scripts.

## Data Availability

Individual-level data from HUNT can be accessed by, or in collaboration with, a Norwegian principal investigator. Researchers can apply for HUNT data access from the HUNT Research Centre (https://www.ntnu.edu/hunt). To do this they must have obtained project approval from the Regional Committee for Medical and Health Research Ethics (REC)^26^. Information on the application and conditions for data access in HUNT is available at https://www.ntnu.edu/hunt/data. Qualified investigators may access the CHS data by following the study policies described at https://chs-nhlbi.org/CHS_DistribPolicy. The authors are restricted from sharing CHS data as per the terms of their data-use agreement. Access to the UK Biobank data can be obtained by application to the UK Biobank (https://www.ukbiobank.ac.uk/). All other data supporting the findings of this study are available from the corresponding author upon reasonable request.

## Code availability

Analyses have been performed using R v.4.1.1 (https://cran.r-project.org/) and the R packages survival (v.3.2-13) for Cox regressions; glmnet (v.4.1-8) for LASSO regression; pROC (v.1.18.5), ROCR (v.1.0-11), PredictABEL (v.1.2-4) for AUC and ROC analyses; Hmisc (v.5.1-2), compareC for C-index calculation and testing; and survminer (v.0.4.9), rms (v.6.8-0) and metafor (v.4.4-0) for plots. The FRAX tool is available at https://www.sheffield.ac.uk/FRAX/ (v.1.4.4). Custom written R scripts are available at https://github.com/marianethander/Protein_Risk_Score and are included in the Supplementary Code file.
