# Publisher Correction: A plasma protein-based risk score to predict hip fractures

**DOI:** 10.1038/s43587-024-00717-w

**Published:** 2024-09-10

**Authors:** Thomas R. Austin, Maria Nethander, Howard A. Fink, Anna E. Törnqvist, Diana I. Jalal, Petra Buzkova, Joshua I. Barzilay, Laura Carbone, Maiken E. Gabrielsen, Louise Grahnemo, Tianyuan Lu, Kristian Hveem, Christian Jonasson, Jorge R. Kizer, Arnulf Langhammer, Kenneth J. Mukamal, Robert E. Gerszten, Bruce M. Psaty, John A. Robbins, Yan V. Sun, Anne Heidi Skogholt, John A. Kanis, Helena Johansson, Bjørn Olav Åsvold, Rodrigo J. Valderrabano, Jie Zheng, J. Brent Richards, Eivind Coward, Claes Ohlsson

**Affiliations:** 1 Cardiovascular Health Research Unit, University of Washington, Seattle, WA US; 2 Department of Internal Medicine and Clinical Nutrition, Institute of Medicine, Sahlgrenska Osteoporosis Centre, Centre for Bone and Arthritis Research at the Sahlgrenska Academy, University of Gothenburg, Gothenburg, Sweden; 3 Bioinformatics and Data Center, Sahlgrenska Academy, University of Gothenburg, Gothenburg, Sweden; 4 Geriatric Research Education and Clinical Center, VA Health Care System, Minneapolis, MN US; 5 Department of Medicine, University of Minnesota, Minneapolis, MN US; 6 Division of Nephrology, Department of Internal Medicine, Carver College of Medicine, Iowa City, IA US; 7 Iowa City VA Medical Center, Iowa City, IA US; 8 Department of Biostatistics, University of Washington, Seattle, WA US; 9 Division of Endocrinology, Kaiser Permanente of Georgia, Atlanta, GA US; 10 Charlie Norwood VAMC, Augusta, GA US; 11 Division of Rheumatology, Department of Medicine, Medical College of Georgia, Augusta University, Augusta, GA US; 12 HUNT Center for Molecular and Clinical Epidemiology, Department of Public Health and Nursing, Norwegian University of Science and Technology, Trondheim, Norway; 13 Lady Davis Institute for Medical Research, Jewish General Hospital, Montreal, Quebec Canada; 14 Quantitative Life Sciences Program, McGill University, Montreal, Quebec Canada; 15 5 Prime Sciences Inc, Montreal, Quebec Canada; 16 HUNT Research Centre, NTNU, Levanger, Norway; 17 Levanger Hospital, Nord-Trøndelag Hospital Trust, Levanger, Norway; 18 Cardiology Section, San Francisco VA Health Care System, San Francisco, CA US; 19 Department of Medicine, Epidemiology and Biostatistics, University of California San Francisco, San Francisco, CA US; 20 Department of Medicine, Beth Israel Deaconess Medical Center, Brookline, MA US; 21 Departments of Medicine, Epidemiology, and Health Systems and Population Health, University of Washington, Seattle, WA US; 22 Department of Medicine, University of California, Davis, CA US; 23 Department of Epidemiology, Rollins School of Public Health, Emory University, Atlanta, GA US; 24 Centre for Metabolic Bone Diseases, University of Sheffield Medical School, Sheffield, UK; 25 Mary McKillop Institute for Health Research, Australian Catholic University, Melbourne, Victoria Australia; 26 Department of Endocrinology, Clinic of Medicine, St. Olavs Hospital, Trondheim University Hospital, Trondheim, Norway; 27 Research Program in Men’s Health, Aging and Metabolism, Brigham and Women’s Hospital, Harvard Medical School, Boston, MA US; 28 Department of Endocrine and Metabolic Diseases, Shanghai Institute of Endocrine and Metabolic Diseases, Ruijin Hospital, Shanghai Jiao Tong University School of Medicine, Shanghai, China; 29 Shanghai National Clinical Research Center for metabolic Diseases, Key Laboratory for Endocrine and Metabolic Diseases of the National Health Commission of the PR China, Shanghai Key Laboratory for Endocrine Tumor, Shanghai Digital Medicine Innovation Center, Ruijin Hospital, Shanghai Jiao Tong University School of Medicine, Shanghai, China; 30 MRC Integrative Epidemiology Unit (IEU), Bristol Medical School, University of Bristol, Oakfield House, Oakfield Grove, Bristol, UK; 31 Department of Human Genetics, McGill University, Montreal, Quebec Canada; 32 Department of Epidemiology, Biostatistics, and Occupational Health, McGill University, Montreal, Quebec Canada; 33 Department of Medicine, McGill University, Montreal, Quebec Canada; 34 Department of Twin Research, King’s College London, London, UK; 35 Region Västra Götaland, Sahlgrenska University Hospital, Department of Drug Treatment, Gothenburg, Sweden

**Keywords:** Predictive markers, Predictive markers

Correction to: *Nature Aging* 10.1038/s43587-024-00639-7, published online 27 May 2024.

In the version of this article initially published, there was an error in Table 1 row “UK Biobank-Olink, randomly selected / Proteomic risk score”. The *P* value shown as “1.6 × 10^–4^” has been corrected to “1.6 × 10^–24^” in the HTML and PDF versions of the article.

