# Supplementary material for: A plasma protein-based risk score to predict hip fractures
Source: Nat Aging. 2024 May 27;4(8):1064–75. doi: 10.1038/s43587-024-00639-7 (PMC11333168; doi:10.1038/s43587-024-00639-7)
Supplement: Supplementary file 2 — Reporting Summary [file 43587_2024_639_MOESM2_ESM.pdf]

nature portfolio

Corresponding author(s): Claes Ohlsson
Last updated by author(s): Apr 20, 2024

Reporting Summary

Nature Portfolio wishes to improve the reproducibility of the work that we publish. This form provides structure for consistency and transparency in reporting. For further information on Nature Portfolio policies, see our Editorial Policies and the Editorial Policy Checklist.

Statistics

For all statistical analyses, confirm that the following items are present in the figure legend, table legend, main text, or Methods section.

n/a	Confirmed	
☐	☒	The exact sample size (*n*) for each experimental group/condition, given as a discrete number and unit of measurement
☒	☐	A statement on whether measurements were taken from distinct samples or whether the same sample was measured repeatedly
☐	☒	The statistical test(s) used AND whether they are one- or two-sided<br>*Only common tests should be described solely by name; describe more complex techniques in the Methods section.*
☐	☒	A description of all covariates tested
☐	☒	A description of any assumptions or corrections, such as tests of normality and adjustment for multiple comparisons
☐	☒	A full description of the statistical parameters including central tendency (e.g. means) or other basic estimates (e.g. regression coefficient) AND variation (e.g. standard deviation) or associated estimates of uncertainty (e.g. confidence intervals)
☐	☒	For null hypothesis testing, the test statistic (e.g. *F*, *t*, *r*) with confidence intervals, effect sizes, degrees of freedom and *P* value noted<br>*Give P values as exact values whenever suitable.*
☒	☐	For Bayesian analysis, information on the choice of priors and Markov chain Monte Carlo settings
☒	☐	For hierarchical and complex designs, identification of the appropriate level for tests and full reporting of outcomes
☐	☒	Estimates of effect sizes (e.g. Cohen's *d*, Pearson's *r*), indicating how they were calculated

Our web collection on statistics for biologists contains articles on many of the points above.

Software and code

Policy information about availability of computer code

Data collection	The SomaScan 5K version 4.0 (CHS cohort and HUNT-SomaScan-5K cohort)<br>SomaScan 7K version 4.1 (HUNT-SomaScan-7K cohort)<br>Olink Explore 1536 platform.
Data analysis	UKB RAP, R v4.1.1 (https://cran.r-project.org/)<br>The following R packages were used:<br>survival v3.2-13 for Cox regressions,<br>glmnet v4.1-8 for LASSO regression,<br>pROC v1.18.5, ROCR v1.0-11, PredictABEL v1.2-4 for AUC and ROC analyses,<br>Hmisc v5.1-2, compareC v1.3.2 for C-index calculation and testing,<br>metafor v4.4-0 for meta analysis<br>survminer v0.4.9, rms v6.8-0 and forestplot v3.1.3 for plots,<br>FRAX tool (https://www.sheffield.ac.uk/FRAX/; version 1.4.4.)

For manuscripts utilizing custom algorithms or software that are central to the research but not yet described in published literature, software must be made available to editors and reviewers. We strongly encourage code deposition in a community repository (e.g. GitHub). See the Nature Portfolio guidelines for submitting code & software for further information.

Data

Policy information about availability of data

All manuscripts must include a data availability statement. This statement should provide the following information, where applicable:

- Accession codes, unique identifiers, or web links for publicly available datasets
- A description of any restrictions on data availability
- For clinical datasets or third party data, please ensure that the statement adheres to our policy

Individual level data from HUNT can be accessed by, or in collaboration with, a Norwegian principal investigator. Researchers can apply for HUNT data access from HUNT Research Centre (https://www.ntnu.edu/hunt). To do this they must have obtained project approval from the Regional Committee for Medical and Health Research Ethics (REC). Information on the application and conditions for data access is available at https://www.ntnu.edu/hunt/data.
Qualified investigators may access CHS data by following study policies as described here: https://chs-nhlbi.org/CHS_DistribPolicy. The authors are restricted from sharing CHS data per the terms of their data use agreement.
Access to UK biobank data can be obtained by application to UK biobank (https://www.ukbiobank.ac.uk/). All other data supporting the findings of this study are available from the corresponding author upon reasonable request.

Research involving human participants, their data, or biological material

Policy information about studies with human participants or human data. See also policy information about sex, gender (identity/presentation), and sexual orientation and race, ethnicity and racism.

Reporting on sex and gender	Since the risk of fracture differs between males and females, all analyses have been either adjusted for or stratified by self-reported sex. In CHS and UK Biobank, gender was determined by self-report at baseline examination. In HUNT, sex was used and determined by genetics. The data of the large well-powered UK Biobank are given gender stratified (Table S8). In addition, descriptives data are given gender (CHS and UK Biobank) or sex (HUNT) stratified in Table S1. All other association analyses are adjusted for gender (CHS and UK Biobank) or sex (HUNT).
Reporting on race, ethnicity, or other socially relevant groupings	All analyses including CHS or UKB participants have been adjusted for self-reported ethnicity.
Population characteristics	UK Biobank is a population-based cohort of approximately 500,000 participants aged 37-73 years.<br>CHS is a population-based longitudinal study of coronary heart disease and stroke in adults aged 65 years and older.<br>HUNT comprises data and samples obtained through four population surveys between 1984 and 2019 in 230,000 people from the Norwegian county of Trøndelag.<br>See details in Supplementary Table 1 for the four included cohorts
Recruitment	UK Biobank participants were recruited between 2006 and 2010.<br>CHS participants were recruited from four U.S. communities: Forsyth County, North Carolina; Sacramento County, California; Washington County, Maryland; and Pittsburgh, Pennsylvania. At baseline (1989-1990), 5,201 individuals were enrolled.<br>HUNT: All people living in the county of Nord-Trøndelag, Mid-Norway, that would pass the age of 20 during the period the field stations were in their municipality were eligible to participate in HUNT. Eligible participants, identified through the Norwegian National Population Register, were invited to HUNT by the HUNT research center, and if needed, they were reminded once.<br>For details, see the methods section.
Ethics oversight	The CHS study was approved by IRBs at each of the 4 field centers and the Coordinating Center. CHS is currently under a single IRB at the University of Washington (current approval number MODCR00000825). All CHS participants provided written informed consent.<br>The HUNT study has ethical approval from the Regional Committee for Medical and Health Research Ethics (REK Central Norway 2015/615) and informed consent was obtained from all participants.<br>The UK Biobank has ethical approval from the North West Multi-centre Research Ethics Committee (North West Research Ethics Committee (11/NW/0382), and informed consent was obtained from all participants. The present research was approved by the UK Biobank Research and Access Committee (application number 51784).

Note that full information on the approval of the study protocol must also be provided in the manuscript.

Field-specific reporting

Please select the one below that is the best fit for your research. If you are not sure, read the appropriate sections before making your selection.

☒ Life sciences ☐ Behavioural & social sciences ☐ Ecological, evolutionary & environmental sciences

For a reference copy of the document with all sections, see nature.com/documents/nr-reporting-summary-flat.pdf

Life sciences study design

All studies must disclose on these points even when the disclosure is negative.

Sample size	For the derivation of Proteomic risk scores, participants from CHS were included. In total 456 incident hip fracture cases and 2,715 controls. Validation cohorts included HUNT-SomaScan-5K (187 incident hip fracture cases and 3,072 controls), HUNT-SomaScan-7K (155 incident hip fracture cases and 1,833 controls) and UK-Biobank (686 incident hip fracture cases and 50,190 controls). Sample sizes for both discovery and replication were chosen on the basis of all data available at the time for analysis. No statistical method was used to predetermine sample size.
Data exclusions	To reduce potential selection bias, participants selected for the UKB Covid-19 study were excluded from this study as they were not randomized into the subset used in proteomic studies.
Replication	Validation of the proteomic risk score were performed in 3 cohorts (described above). All replications using the weighted proteomic risk score were successful while the replications in the HUNT validation cohort of the two proteomic risk scores based on machine learning were less convincing as described in the article.
Randomization	Not applicable (It is population-based longitudinal cohort study and not a randomized clinical trial)
Blinding	Not applicable (It is population-based longitudinal cohort study and not a randomized clinical trial)

Reporting for specific materials, systems and methods

We require information from authors about some types of materials, experimental systems and methods used in many studies. Here, indicate whether each material, system or method listed is relevant to your study. If you are not sure if a list item applies to your research, read the appropriate section before selecting a response.

Materials & experimental systems

n/a	Involved in the study	
☒	☐	Antibodies
☒	☐	Eukaryotic cell lines
☒	☐	Palaeontology and archaeology
☒	☐	Animals and other organisms
☒	☐	Clinical data
☒	☐	Dual use research of concern
☒	☐	Plants

Methods

n/a	Involved in the study	
☒	☐	ChIP-seq
☒	☐	Flow cytometry
☒	☐	MRI-based neuroimaging

Plants

Seed stocks	NA
Novel plant genotypes	NA
Authentication	NA
