# Supplementary material for: A plasma protein-based risk score to predict hip fractures
Source: Nat Aging. 2024 May 27;4(8):1064–75. doi: 10.1038/s43587-024-00639-7 (PMC11333168; doi:10.1038/s43587-024-00639-7)
Supplement: Supplementary file 4 — Custom written scripts. [file 43587_2024_639_MOESM4_ESM.docx]

# IDI.R

#Maria Nethander 2014-03-26

IDI<-function(basemodel,riskmodel,events,nonevents){

#basemodel and riskmodel are the output from a glm()

#events and nonevents are vectors with the row numbers of events/nonevents in the data frame used in the glm for the base and risk models

predbase = predRisk(basemodel)

predrisk = predRisk(riskmodel)

#Mean risk for base model

ev_prob_base = predbase[events]

non_ev_prob_base = predbase[-events]

IDI_event_base = mean(ev_prob_base)

IDI_non_event_base = mean(non_ev_prob_base)

#Mean risk for risk model

ev_prob_risk = predrisk[events]

non_ev_prob_risk = predrisk[-events]

IDI_event_risk = mean(ev_prob_risk)

IDI_non_event_risk = mean(non_ev_prob_risk)

#IDI

IDI_ev<-IDI_event_risk-IDI_event_base

IDI_nonev<-IDI_non_event_base-IDI_non_event_risk

IDI<-IDI_ev+IDI_nonev

#SE

SE_ev<-sd(predrisk[events] - predbase[events])/sqrt(length(events))

SE_nonev<-sd(predrisk[nonevents] - predbase[nonevents])/sqrt(length(nonevents))

SE<-sqrt(SE_ev^2+SE_nonev^2)

#Z

z_ev<-IDI_ev/SE_ev

z_nonev<-IDI_nonev/SE_nonev

z<-IDI/SE

#p

p_ev = 2 * (1 - pnorm(abs(z_ev)))

p_nonev = 2 * (1 - pnorm(abs(z_nonev)))

p = 2 * (1 - pnorm(abs(z)))

result<-list(IDI=IDI, SE=SE, Z=z, p=p, IDI_event=IDI_ev, SE_event=SE_ev, Z_event=z_ev, p_event=p_ev, IDI_nonevent=IDI_nonev, SE_nonevent=SE_nonev, Z_nonevent=z_nonev, p_nonevent=p_nonev)

return(result)

}

# NRI.R

###This function calculates continous NRI according to the method described by Pencina et al in "Evaluating the added predictive ability of a new marker: From area under the ROC curve to reclassification and beyond" and "Extensions of net reclassification improvement calculations to measure usefulness of new biomarkers".

#Maria Nethander

#2014-03-26

NRI<-function(basemodel,riskmodel,events,nonevents){

#basemodel and riskmodel are the output from a glm()

#events and nonevents are vectors with the row numbers of events/nonevents in the data frame used in the glm for the base and risk models

predbase = predRisk(basemodel)

predrisk = predRisk(riskmodel)

p_up_ev<-length(which((predbase[events]-predrisk[events])<0))/length(events)

p_down_ev<-length(which((predbase[events]-predrisk[events])>0))/length(events)

p_up_nonev<-length(which((predbase[-events]-predrisk[-events])<0))/length(nonevents)

p_down_nonev<-length(which((predbase[-events]-predrisk[-events])>0))/length(nonevents)

NRI_ev<-p_up_ev-p_down_ev

NRI_nonev<-p_down_nonev-p_up_nonev

NRI<-NRI_ev+NRI_nonev

SE_ev<-sqrt((p_up_ev+p_down_ev)/length(events) - (p_up_ev-p_down_ev)^2/length(events))

SE_nonev<-sqrt((p_up_nonev+p_down_nonev)/length(nonevents) - (p_down_nonev-p_up_nonev)^2/length(nonevents))

SE<-sqrt(SE_ev^2+SE_nonev^2)

z_ev<-NRI_ev/SE_ev

z_nonev<-NRI_nonev/SE_nonev

z<-NRI/SE

p_ev<- 2 * (1 - pnorm(abs(z_ev)))

p_nonev<- 2 * (1 - pnorm(abs(z_nonev)))

p<- 2 * (1 - pnorm(abs(z)))

result<-list(NRI=NRI, SE=SE, Z=z, p=p, NRI_event=NRI_ev, SE_event=SE_ev, Z_event=z_ev, p_event=p_ev, NRI_nonevent=NRI_nonev, SE_nonevent=SE_nonev, Z_nonevent=z_nonev, p_nonevent=p_nonev)

return(result)

}

# NRIcutoff.R

###This function calculates continous NRI according to the method described by Pencina et al in "Evaluating the added predictive ability of a new marker: From area under the ROC curve to reclassification and beyond" and "Extensions of net reclassification improvement calculations to measure usefulness of new biomarkers".

#Maria Nethander, Bioinformatics Core Facility, Gothenburg

#2014-03-26

NRIcutoff<-function(basemodel,riskmodel,events,nonevents,cutoff){

#basemodel and riskmodel are the output from a glm()

#events and nonevents are vectors with the row numbers of events/nonevents in the data frame used in the glm for the base and risk models

predbase = predRisk(basemodel)

predrisk = predRisk(riskmodel)

predbase = as.numeric(predbase>=cutoff)

predrisk = as.numeric(predrisk>=cutoff)

p_up_ev<-length(which((predbase[events]-predrisk[events])<0))/length(events)

p_down_ev<-length(which((predbase[events]-predrisk[events])>0))/length(events)

p_up_nonev<-length(which((predbase[-events]-predrisk[-events])<0))/length(nonevents)

p_down_nonev<-length(which((predbase[-events]-predrisk[-events])>0))/length(nonevents)

NRI_ev<-p_up_ev-p_down_ev

NRI_nonev<-p_down_nonev-p_up_nonev

NRI<-NRI_ev+NRI_nonev

SE_ev<-sqrt((p_up_ev+p_down_ev)/length(events) - (p_up_ev-p_down_ev)^2/length(events))

SE_nonev<-sqrt((p_up_nonev+p_down_nonev)/length(nonevents) - (p_down_nonev-p_up_nonev)^2/length(nonevents))

SE<-sqrt(SE_ev^2+SE_nonev^2)

z_ev<-NRI_ev/SE_ev

z_nonev<-NRI_nonev/SE_nonev

z<-NRI/SE

p_ev<- 2 * (1 - pnorm(abs(z_ev)))

p_nonev<- 2 * (1 - pnorm(abs(z_nonev)))

p<- 2 * (1 - pnorm(abs(z)))

result<-list(NRI=NRI, SE=SE, Z=z, p=p, NRI_event=NRI_ev, SE_event=SE_ev, Z_event=z_ev, p_event=p_ev, NRI_nonevent=NRI_nonev, SE_nonevent=SE_nonev, Z_nonevent=z_nonev, p_nonevent=p_nonev)

return(result)

}

# test2AUC.R

#Maria Nethander 2014-03-26

#MN added confidence intervals for AUC. 2019-01-30

#MN added possibility to use one sided tests. 2019-03-19

library("ROCR")

library(foreign)

library(pROC)

library(PredictABEL)

library(Hmisc)

test2AUC<-function(basemodel,riskmodel,data, response,doPlot=F, filename="ROCplot",alternative="two.sided"){

#Basemodel

predbase=predict(basemodel, newdata=data, type="response") # i newdata anger man var den ska leta efter x-variablerna som

predbase2=prediction(predbase, response)

perfbase=performance(predbase2, "tpr", "fpr")

#Riskmodel

predrisk=predict(riskmodel, newdata=data, type="response")

predrisk2=prediction(predrisk, response)

perfrisk=performance(predrisk2, "tpr", "fpr")

#Statistical test

rocbase=roc(response, predbase,ci=T)

rocrisk=roc(response, predrisk,ci=T)

test<-roc.test(rocbase, rocrisk, reuse.auc=T,alternative="two.sided")

#Results

aucbase<-test$estimate[1]

aucrisk<-test$estimate[2]

cibase<-rocbase$ci

cirisk<-rocrisk$ci

Z<-test$statistic

p<-test$p.value

result<-list(aucbase,cibase,aucrisk,cirisk,Z,p)

## result<-list(AUCbasemodel=aucbase,AUC95CIbasemodel=cibase,AUCriskmodel=aucrisk,AUC95CIriskmodel=cirisk,Z=Z,p=p)

## result<-c(aucbase,cibase,aucrisk,cirisk,Z,p)

## names(result)<-c("AUC for basemodel","AUC 95CI for basemodel","AUC for riskmodel","AUC 95CI for riskmodel","Z","p")

#Plot

if(doPlot){

pdf(paste(filename,".pdf",sep=""))

plot(perfbase)

plot(perfrisk, avg="vertical", lwd=3, col="red", spread.estimate="stderror",plotCI.lwd=2,add=TRUE, main="Hip")

abline(coef=c(0,1), lwd=3)

plot(perfbase, col="blue", lwd=3, add=T )

dev.off()

}#endif

return(result)

}
